# From eschar to diagnosis: A case report on scrub typhus causing multi-organ failure in a returning traveller

**DOI:** 10.1016/j.clinme.2025.100307

**Published:** 2025-04-01

**Authors:** Patrick Eaton, Ahmed Ahmed, Emyr Huws

**Affiliations:** a ACCS 3–Ysbyty Gwynedd, Betsi Cadwalader University Health Board, Bangor, Gwynedd, Wales; b IMT 2–Ysbyty Gwynedd, Betsi Cadwalader University Health Board, Bangor, Gwynedd, Wales; c Consultant Anaesthetist, Consultant in Anaesthesia and Intensive Care Medicine, Ysbyty Gwynedd, Betsi Cadwalader University Health Board, Bangor, Gwynedd, Wales

**Keywords:** *Orientia tsutsugamushi*, Scrub typhus, Rickettsial infection, Tropical infection in travellers, Imported pathogens

## Abstract

Scrub typhus, caused by *Orientia tsutsugamushi*, is a rickettsial infection transmitted by mite bites, often underdiagnosed in travellers from endemic regions. This case report describes a 65-year-old female with hypertension who developed severe scrub typhus after a trip to Sri Lanka. She presented with fever, myalgia, headache, fatigue and a scabbed lesion. Initial tests showed neutrophilia, lymphocytopenia and elevated liver enzymes. Malaria was ruled out, and empiric treatment with intravenous Tazocin was initiated. On day 5, she developed *Clostridium difficile* infection, requiring a switch to oral vancomycin. By day 5, her condition worsened with hypoxia, hypotension, oliguria and renal failure. Chest X-ray revealed bilateral infiltrates and subsequently, she was transferred to critical care. Tests showed positive IgM test for *O. tsutsugamushi*. Oral doxycycline was started, resulting in rapid improvement. PCR confirmed scrub typhus. This case underscores the importance of early diagnosis and treatment with doxycycline in travellers from endemic areas presenting with febrile illness.

## Introduction

Rickettsial diseases are important causes of morbidity and mortality worldwide. They are often misdiagnosed and the true burden is underestimated. A recent systematic review of imported infections to the UK demonstrated these pathogens represent a major cause of non-malarial fever.^1^ Timely recognition and treatment with doxycycline, before testing, is crucial to avoid life-threatening complications.^1^

We report a case of scrub typhus, a severe and debilitating illness caused by *Orientia tsutsugamushi*, a bacterium from the family *Rickettsiaceae*, in a traveller returning from a high-risk area.

## Case report

**Image 1:**
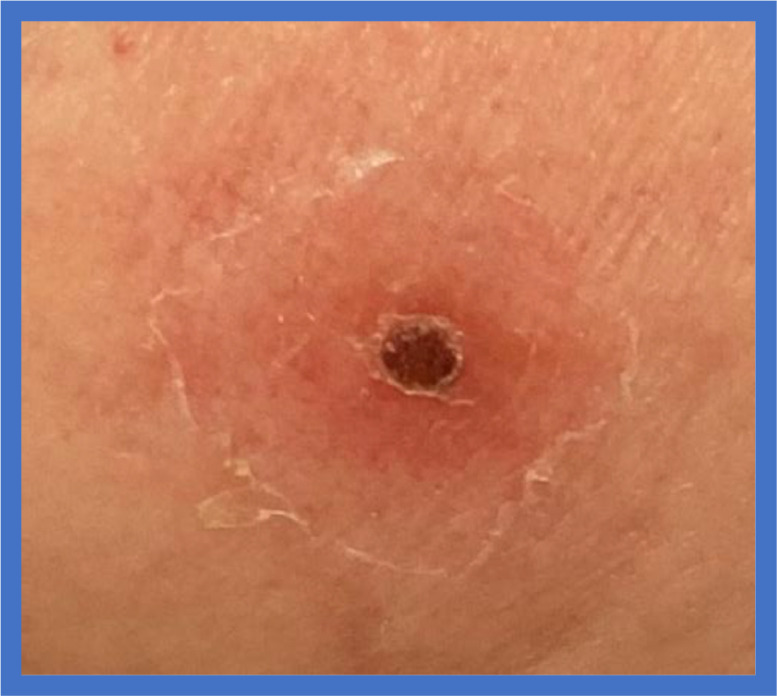
Illustrating an eschar lesion under patient’s left breast.

A 65-year-old female patient presented with a 4-day history of fever, myalgia, headache, fatigue, constipation and cough. The patient had returned from a bicycle touring trip to Sri Lanka 17 days ago. She had a background of treated hypertension. Examination revealed tachycardia (108 bpm) and fever (39.4 °C). A small scabbed lesion was found beneath the patient’s left breast (Image 1); however, she denied any history of insect bites. No other rashes were noted.

There were no enlarged palpable lymph nodes, hepatomegaly or splenomegaly noted on examination and subsequently, no imaging studies were done to investigate this.

**Image 2:**
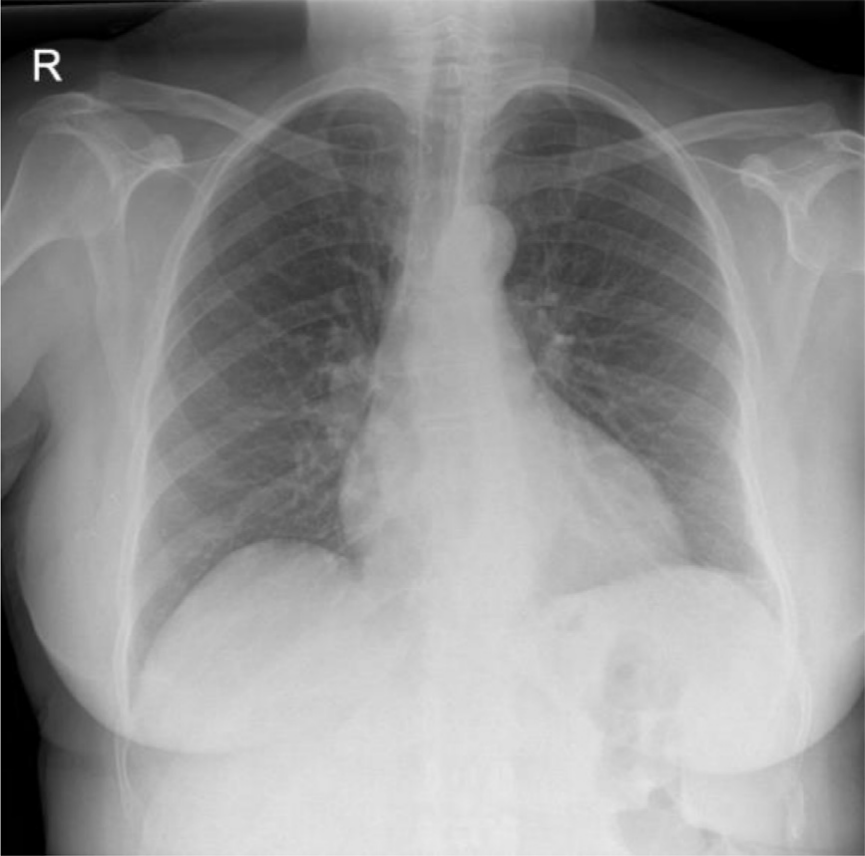
CXR clear.

Initial blood tests revealed neutrophilia, lymphocytopenia, mild transaminitis and raised CRP (261 mg/L). Malaria films were negative. CXR was unremarkable (Image 2). Blood cultures were collected on admission which showed no growth.

Patient was admitted and treated with intravenous fluids and Tazocin (broad-spectrum antibiotic for infection of unknown source as per trust protocol).

A blood sample was sent to the Rare and Imported Pathogens Laboratory (RIPL) in Porton Down for a panel test for ‘selected rare and imported pathogens’.

On day 3, the patient developed watery diarrhoea. She remained febrile with static inflammatory markers. Stool sample was positive for *C. difficile* DNA and toxin. Tazocin was stopped and oral vancomycin commenced.

**Image 3:**
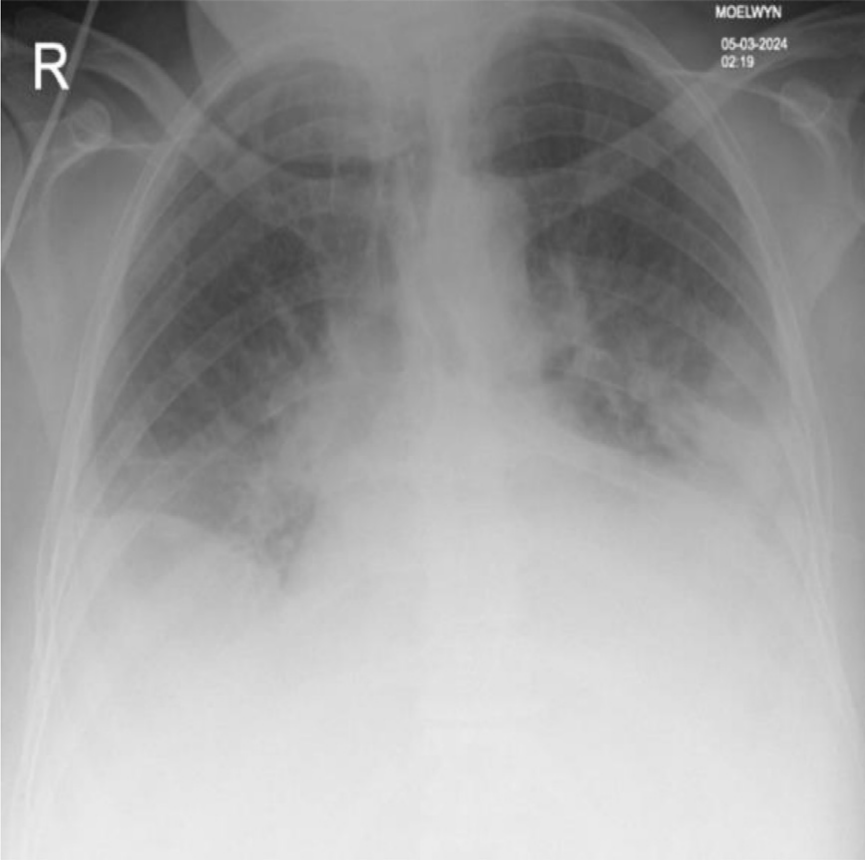
CXR showing bilateral interstitial infiltrates.

On day 5, the patient developed a rapidly escalating oxygen requirement to 15 L through a reservoir mask; in addition to hypotension, oliguria and confusion. CXR showed extensive bilateral infiltrations (Image 3). She was found to have worsening neutrophilia, thrombocytopenia, transaminitis and acute renal failure (creatinine 256 µmol/L on a baseline of 70 µmol/L). Troponin was not tested as was not clinically indicated (Table 1).

**Table 1:** Blood results progress during admission.

Test	Day 1	Day 5
CRP	261	277
ALT	65	95
Creatinine (eGFR)	76 µmol/L (66 mL/min/1.73 m^2^)	256 µmol/L (16 mL/min/1.73 m^2^)
WCC (neutrophils)	12.8 × 10^9^/L (11.9 × 10^9^/L)	22.7 × 10^9^/L (17.5 × 10^9^/L)
Platelets	140 × 10^9^/L	73 × 10^9^/L
Lactate	1.3	2.5

The patient was admitted to critical care for supportive treatment including high-flow oxygen, intermittent continuous positive airway pressure ventilation as tolerated, and a noradrenaline infusion. Arterial blood gas showed type 1 respiratory failure and metabolic acidosis with pH 7.24 and base excess -13. Further blood cultures were collected at day 5 and day 6 respectively, which were negative.

On admission to critical care, the ‘selected rare and imported pathogens’ panel was available for review and demonstrated IgM positive for *O. tsutsugamushi* with indeterminate results for IgG. The patient was started on oral doxycycline and began to improve after 24 h. There was no further requirement of high-flow oxygen or vasoactive support after 48 h, and the patient was safely stepped down to the ward. Subsequent blood PCR testing confirmed the presence of *O. tsutsugamushi*, with the detection of positive IgM antibodies and IgG antibodies, provided a definitive diagnosis of scrub typhus.

## Discussion

This case highlights the considerable diagnostic difficulties associated with scrub typhus, an infectious disease that, alongside other rickettsial illnesses, is seeing a resurgence in global incidence.^1^

Rickettsial diseases primarily affect travellers returning from endemic regions within the ‘Tsutsugamushi triangle’ of the Asia-Pacific, which spans from northern Japan and far eastern Russia to northern Australia in the south and Pakistan in the west. However, recent reports indicate its occurrence in areas outside this traditional geographic range, supporting evidence of an increasing global prevalence of the disease.^2^^,^^3^ As one of the top five acute, potentially fatal diseases in travellers from tropical areas, rickettsial diseases pose a serious public health concern.^2^ Each year, approximately one million cases are reported worldwide, with untreated cases showing a fatality rate as high as 30%.^3^

We will focus our discussion now on scrub typhus as a diagnosis for this case report. Scrub typhus is marked by systemic vasculitis, causing vascular damage in organs such as the skin, liver, brain and lungs. The bacteria multiply at the inoculation site, leading to the formation of an eschar and triggering rickettsaemia, which infects the endothelium causing fluid leaks giving the pictures of various complications of the disease as will be discussed.^4^

The infection reservoirs for scrub typhus include chiggers (larvae of trombiculid mites) and rats, with humans becoming accidental hosts. Transmission occurs through trombiculid mites, commonly found in areas with long grasses or dirt-floor homes.^4^

The clinical presentation of scrub typhus varies, but the incubation period generally ranges from 6 to 14 days. Patients commonly experience symptoms such as fever, myalgia, headache, dry cough and, in some cases, a diffuse rash. A key diagnostic marker of scrub typhus is the presence of one or more painless eschars at the bite site.^1^ A 2024 systematic review and meta-analysis, encompassing 181 studies and 148,251 samples, reported a 30.34% prevalence of eschar among positive cases, further underscoring its diagnostic importance.^3^

The disease can lead to severe multisystemic complications, including pneumonia, acute respiratory distress syndrome (ARDS)-like symptoms, pulmonary oedema, myocarditis, encephalitis, hepatitis, disseminated intravascular coagulation (DIC), haemophagocytic syndrome and acute kidney injury.^4^ In one study involving 116 patients with severe scrub typhus admitted to intensive care, 96.6% experienced respiratory failure, with 87.9% requiring ventilatory support, and a mortality of 24.1% was reported among these patients.^4^ These findings highlight the severity of scrub typhus in critically ill patients and the significant burden of complications associated with the disease.

Various antibiotics have been studied and compared to doxycycline for treating scrub typhus, particularly in terms of their cure rates and the occurrence of complications. A 2022 network meta-analysis of 14 randomised trials with 1,264 participants found no antibiotic significantly outperformed doxycycline in overall scrub typhus cure rates. However, chloramphenicol had the highest cure rate in paediatric patients, while minocycline was most effective in adults. Second-generation quinolones (eg ofloxacin, ciprofloxacin) and chloramphenicol caused fewer adverse events than doxycycline.^5^

This case reinforces the need for heightened awareness of scrub typhus as a differential diagnosis for febrile illness in patients with travel histories to endemic areas, as well as the importance of early recognition and management to prevent serious complications and reduce mortality.

## Conclusion

In summary, scrub typhus is a vector-borne disease transmitted through the bite of infected mite larvae.^3^ The presence of an eschar, as seen under this patient’s left breast, is a highly specific clinical indicator of the infection.^4^ Diagnosis of rickettsial infections in UK, including scrub typhus, can be confirmed through serology and PCR testing available at the Rare and Imported Pathogens Laboratory (RIPL).^1^ In suspected cases, early treatment with doxycycline, before the results of the selected rare and imported pathogens panel are back, is recommended, particularly if the patient does not respond to standard treatments for common infections. Additionally, the Imported Fever Service offers 24 h expert consultation for clinicians managing cases of suspected scrub typhus.^2^

## Funding

This research did not receive any specific grant from funding agencies in the public, commercial, or not-for-profit sectors.

## Consent for publication

Written informed consent has been obtained from the patient for the publication of this case report, including any accompanying images or identifiable information. The patient has been fully informed about the purpose of the publication, the potential use of their case details in scientific, educational and promotional materials, and the irrevocable nature of their consent once the report is published. All efforts have been made to protect the patient’s anonymity, and any identifying details have been omitted unless essential for scientific purposes. This consent process complies with all applicable data protection and privacy laws, including but not limited to HIPAA, GDPR and other relevant regulations. A copy of the signed consent form is retained by the authors and is available upon request.

## CRediT authorship contribution statement

**Patrick Eaton:** Writing – review & editing, Visualization, Validation, Resources, Methodology, Investigation, Data curation, Conceptualization. **Ahmed Ahmed:** Writing – review & editing, Writing – original draft, Project administration, Investigation, Funding acquisition, Formal analysis, Data curation. **Emyr Huws:** Supervision.

## Declaration of competing interest

The authors declare that they have no known competing financial interests or personal relationships that could have appeared to influence the work reported in this paper.
